# Evaluation of Ultrasound-Based Parameters for the Assessment of Hepatic Steatosis and Fibrosis in Hungarian Wilson’s Disease Patients

**DOI:** 10.3390/diagnostics16101433

**Published:** 2026-05-08

**Authors:** Anikó Folhoffer, Boglárka Zsély, Anna Krolopp, Dániel Németh, Tamás Tóth, Csaba Lőrinczi, Krisztina Hagymási, Anna Egresi, Csenge Bánhidi, Judit Halász, Barbara Csongrády, Bettina Katalin Budai, Róbert Stollmayer, Zsuzsanna Jakab, András Laki, Gabriella Győri, Aladár Dávid Rónaszéki, Pál Maurovich-Horvát, Ferenc Szalay, Pál Novák Kaposi, István Takács

**Affiliations:** 1 Department of Internal Medicine and Oncology, Semmelweis University, H 1083 Budapest, Hungary; 2 Department of Radiology, Medical Imaging Centre, Semmelweis University, H 1082 Budapest, Hungary; 3 Department of Family Medicine, Semmelweis University, H 1092 Budapest, Hungary; 4 National Institute of Oncology, H 1122 Budapest, Hungary; 5 Department of Surgery, Transplantation, and Gastroenterology, Semmelweis University, H 1082 Budapest, Hungary; 6 Department of Pathology, Forensic and Insurance Medicine Semmelweis University, H 1092 Budapest, Hungary; 7 Clinic for Diagnostic and Interventional Radiology (DIR), Heidelberg University Hospital, 69120 Heidelberg, Germany

**Keywords:** Wilson’s disease, steatofibrosis, non-invasive diagnosis, elastography, surveillance

## Abstract

**Background**: Wilson’s disease (WD) is a genetic disorder of copper metabolism with over 600 disease-causing mutations, leading to variable hepatic and neurological symptoms. Early diagnosis and treatment are crucial. To evaluate hepatic involvement, serum scores and non-invasive imaging techniques complement histology. **Methods**: This pilot study assessed the utility of ultrasound-based tissue attenuation imaging (TAI), tissue scatter distribution imaging (TSI), and shear-wave elastography (SWE) for quantifying steatosis and fibrosis in WD. **Results**: Among 131 treated patients, 53 (mean age 40.5 ± 13.1 years, M/F = 35/18) underwent measurements. Based on literature-validated thresholds, 41 patients did not have significant liver fibrosis, 5 patients had moderate (F2) and 4 advanced (F3) fibrosis, while 3 patients had cirrhosis. The LS (liver stiffness) was in moderate correlation with FIB-4 (r = 0.306, *p* < 0.03), NAFLD fibrosis index (r = 0.336, *p* < 0.02), and APRI (r = 0.31, *p* = 0.0857). Among the WD patients, 37 had no steatosis (S0), 14 had mild steatosis (S1), and 2 had intermediate steatosis (S2); none of them had severe steatosis (S3) based on UEFF calculation. LS correlated positively with calculated free copper and negatively with serum ceruloplasmin. Normal-BMI patients exhibited no significant steatosis (R2 = 0. 0065, *p* = 0.966) by ultrasound-estimated fat fraction (UEFF), while those with BMI > 25 kg/m^2^ had increased UEFF correlating with BMI (R2 = 0.288, *p* < 0.015). Over a five-year follow-up using liver elastography, the fibrosis score did not progress significantly in adequately treated patients. **Conclusions**: Ultrasound with artificial intelligence-derived parameters supports the non-invasive evaluation of hepatic steatosis and fibrosis in WD, complementing clinical and laboratory data. However, population-specific liver stiffness thresholds are still needed.

## 1. Introduction

Wilson’s disease (WD) is an autosomal recessive inherited metabolic disorder of copper metabolism first described by Kinnier Wilson in 1912 [1,2,3]. Due to the defect in biliary excretion, excess copper is accumulated mainly in the liver and brain, leading to various clinical presentations, mostly occurring in young adulthood. However, it may present symptomatically at any age. The youngest patient whose treatment was initiated was 3 years old in Hungary. The international literature reported the youngest child with elevated transaminase levels and genetically confirmed WD at the age of eight months [4]. The late presentation of the disease, even after the 6th decade, is also well known [5,6].

The diagnosis is easily overlooked, but if discovered early, effective treatments are available that will prevent or reverse many manifestations of this disorder. There are several chelating agents and zinc salts for medical therapy. Liver transplantation corrects the underlying pathophysiology and can be lifesaving.

Among the genetic diseases, WD is relatively frequent [7,8]. Family screening is important, since the index patient’s siblings have a 25% risk for WD. It is common in closed populations and in consanguineous marriages, and it is more frequent among Chinese and Asian people [9]. The global prevalence has originally been estimated to be 1 case in 30,000 individuals [10,11], while higher fractions of 1:7000 with 2.5% individuals carrying a causative allele have been reported [8,12].

The diagnosis is based on the clinical (hepatological and neuropsychiatric) symptoms, laboratory findings, the genetic results, and the imaging assessment [8,13]. Two disease-causing pathogen mutations confirm the diagnosis. Otherwise, none of the established diagnostic tests of the Leipzig score alone can clearly diagnose or rule out WD. According to the American Association for Study of Liver Diseases (AASLD), WD can be diagnosed without liver biopsy or molecular testing, by using the following criteria: serum ceruloplasmin of less than 0.20 g/L and raised 24 h urine copper excretion that is greater than 100 μg/24 [14]. However, in the presence of two disease-causing mutations, this alone is sufficient for the diagnosis. Thus far, among the identified more than 1200 ATP7B unique variants, a lot of them are pathogenic, causing impairment of ATP7B function and, ultimately, copper accumulation, but there are several benign variations. Therefore, genetic testing alone is not always sufficient to diagnose WD in asymptomatic patients, thus human mutation databases should be used with caution [15].

Since liver morphology is non-specific, and copper histochemistry may lead to both false-negative and false-positive results, the pathologist usually only suspects the disease or assists in its confirmation.

Although the value of molecular genetic testing is limited due to the high number of possible gene mutations, polymerase chain reaction may be helpful in the evaluation of family members of homozygous index patients [16].

WD can appear in any type of liver disease, from mild to severe steatosis, fulminant type liver disease, fibrosis to cirrhosis, and rarely it may even cause hepatocellular carcinoma.

Early changes in the liver in WD are often non-specific and may be minimal, typically including mild hepatocellular damage, characterized by scattered apoptotic hepatocytes and ballooning degeneration and mild portal inflammation.

Copper deposition in the liver during WD varies significantly, from lobule to lobule in early stages and from nodule to nodule in the cirrhotic liver.

Liver biopsy is not routinely required in the diagnostic work-up of Wilson’s disease and is only rarely indicated. In most cases, the diagnosis can be established without histological assessment, by utilizing the Leipzig scoring system. For longitudinal follow-up, non-invasive modalities have largely supplanted liver biopsy due to their practicality and broader applicability.

Concerning the liver involvement for the diagnosis and in everyday patient care during the general check-up, we use imaging assessment, mainly abdominal ultrasound, in addition to routine laboratory tests, and if necessary, CT or MRI scan. The most accurate information can be obtained through a liver biopsy; however, it is rarely needed, since it carries certain risks [17].

The non-invasive radiologic measurements may play a pivotal role during follow-up to monitor disease progression and evaluate the treatment efficacy of current or emerging therapies. Elastography examines tissue deformation caused by mechanical or acoustic waves. The lower the elasticity of the tissue, i.e., the greater the stiffness caused by fibrosis, the greater the speed of wave propagation and the greater the test result expressed in kPa or m/s.

Steatosis could be the consequence of several other overlapping hepatological disorders, like non-alcoholic, or, according to the new nomenclature, metabolic dysfunction-associated steatotic liver disease (MASLD) or autoimmune hepatitis. Tissue attenuation imaging (TAI), and tissue scatter distribution imaging (TSI) could be used successfully to diagnose and estimate the severity of hepatic steatosis in routine clinical practice with conventional ultrasound machines. It is important to evaluate whether it is the progression of Wilson’s disease or concurrent liver disease of other origin. Measuring the copper parameters from the serum and urine with additional routine laboratory (cholesterol, triglyceride, etc.), immune and viral serological markers, or genes predisposing to fatty liver disease could help to differentiate them [18,19]. However, in obscure cases, liver biopsy could be necessary.

Previously, we have assessed the feasibility of non-invasive ultrasound methods for quantification of liver steatosis in patients with MASLD [20]. We compared the magnetic resonance imaging proton density fat fraction (MRI-PDFF) in the same population, and we determined the correlation between TAI, TSI, and MRI-PDFF and used multiple linear regression analysis to identify any association with clinical variables. We also found that the UEFF and linear multivariable models are robust in diagnosing low-grade steatosis. Switching to a non-linear model could enhance the fit to MRI-PDFF in advanced steatosis [21].

In another study, we aimed to evaluate the technical success rate, interobserver reproducibility, and accuracy of shear wave elastography (SWE) in the staging of hepatitis C virus (HCV)-associated liver fibrosis [22]. Reviewing both of our studies, we can say that SWE can be used in chronic liver diseases.

Our current article focuses on the role of the non-invasive radiological tests in the management of WD patients, based on our data of Hungarian Wilson’s disease patients.

## 2. Materials and Methods

### 2.1. Selection of Patients

We aimed to assess the feasibility of ultrasound-based TAI and TSI for quantification of liver steatosis, and SWE for liver stiffness measurement in WD patients in a retrospective study. Our institutional review board approved the study protocol of research ethics, and all procedures and data handling were conducted in accordance with the Declaration of Helsinki. All participants of the study have signed an informed consent before the quantitative ultrasound scan.

Of the approximately 274 diagnosed WD patients in Hungary, about 131 patients regularly attend our hepatological outpatient unit. At each follow-up appointment, patients were examined physically and standard laboratory parameters, including serum copper, serum ceruloplasmin by nephelometry. When the SWE capable scanner became available, SWE examinations were included, but not in all cases. Monitoring of the patients was based on clinical findings, liver function tests, and we calculated free serum copper and 24 h urinary copper excretion.

The patients were diagnosed and treated at our department. The diagnosis was based on the international WD Leipzig score system published in 2003 [13], and each patient had a score of 4 or more points. The study was approved by the Semmelweis University’s Committee of Research Ethics and was conducted in accordance with the Helsinki Declaration. All patients gave written informed consent.

The eligibility criteria to participate in the study included the following:

Aged 18 years or older, referral to an imaging study and either ultrasound or MRI for Wilson’s disease. The participants’ demographic data were collected from a personal survey, and the medical history and laboratory tests were collected from electronic medical reports.

The final cohort included 53 subjects (18 females and 35 males), the mean age of the participants was 40.5 ± 13.1 years.

### 2.2. Quantitative Ultrasound Scans

Between March and October 2024, we examined 53 of these patients using non-invasive ultrasound methods to simultaneously assess hepatic steatosis and fibrosis. Ultrasound examinations of the liver were performed after at least four hours of fasting using a Samsung RS85 Prestige system (Samsung Medison Co., Ltd., Seoul, Republic of Korea) and a CA1-7S convex probe (Samsung Medison Co., Ltd., Seoul, Republic of Korea). The patients were scanned in a supine position with their arms elevated above their heads. All examiners had a minimum of five years of experience in liver ultrasound. Quantitative measurements were taken in the right lobe from an intercostal view in breath-hold at mid-inspiration. Liver stiffness was measured in kilopascals (kPa) using two-dimensional shear wave elastography (2D-SWE, S-Shearwave Imaging^TM^, Samsung Medison Co., Ltd., Seoul, Republic of Korea) as described previously [23]. Only stiffness values with an interquartile range/median ratio < 30% were accepted for further analysis. The patients were classified into five different fibrosis grades (F0–F4) with cut-off values at 2.5 kPa, 7 kPa, 9.5 kPa, and 12.5 kPa, applying the cut-off values reported by Paternostro et al. [24]. It must be emphasized that these cut-off values reported by Paternostro et al. were derived using vibration-controlled transient elastography (VCTE). Hence, these values should be used with appropriate caution when applying them to other elastography modalities.

**Figure 1:**
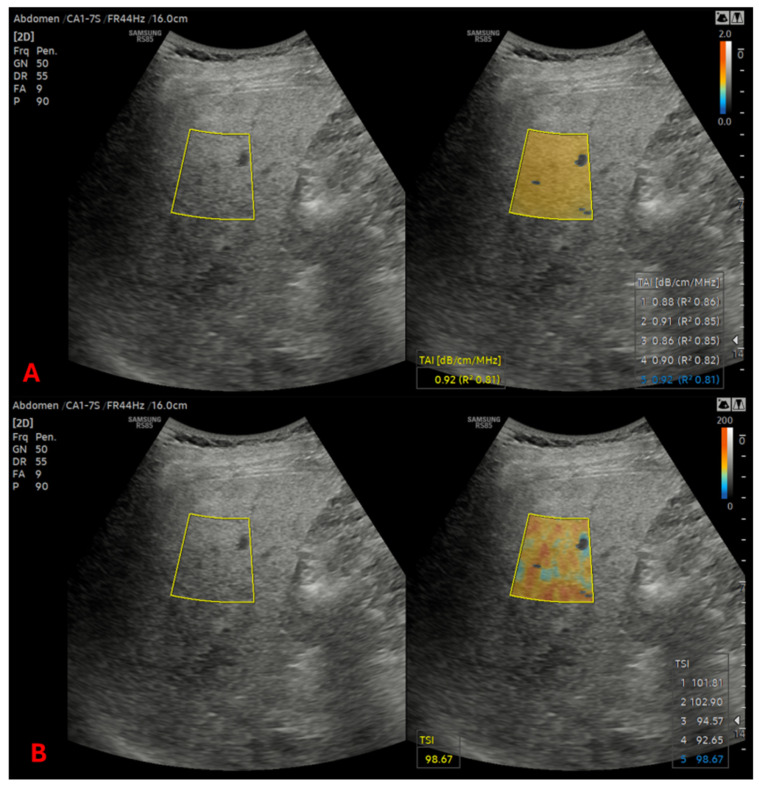
Quantitative ultrasound measurement of liver fat. Tissue attenuation imaging (**A**) and tissue scatter distribution imaging (**B**) of participants with moderate liver fat content of more than 5% less than 15%. Shear wave elastography was applied which ruled out significant fibrosis.

The liver steatosis was assessed using quantitative ultrasound (QUS), for which the Tissue Attenuation Imaging (TAI) and the Tissue Scatter Distribution Imaging (TSI) parameters were measured in the right lobe. The TAI and TSI measurements were repeated 5 times, and the median of the 5 measurements was used for fat quantification (Figure 1). Only TAI values with R2 > 0.6 were considered reliable. The TAI was reported in units of dB/cm/MHz, while the TSI was reported in arbitrary units. The ultrasound-derived fat fraction (UEFF) was calculated as a percentage (%) using a non-linear least-squares model, which has been described in detail in our previous publication [25]. We differentiated between four severity grades of steatosis (S0–S3) among our patients by using cut-off values at 5%, 17% and 22% steatosis [26]. We compared each patient’s previous data with their latest measurements.

### 2.3. Clinical and Laboratory Data Collection

**Figure 2:**
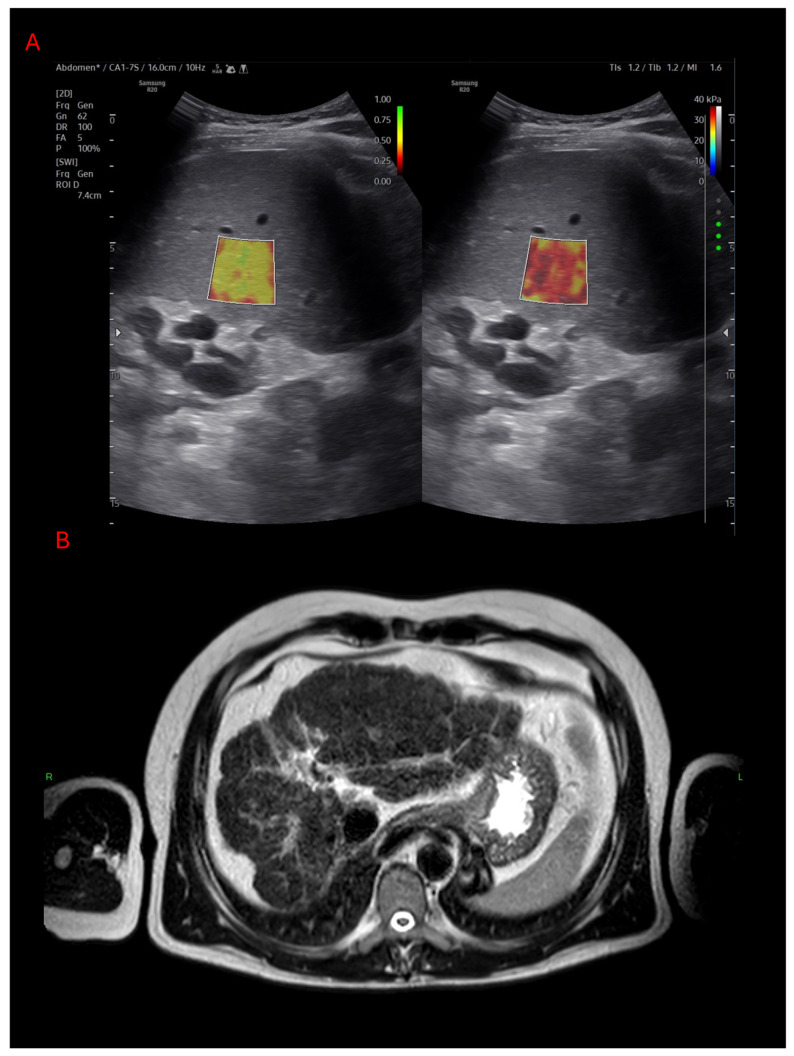
Cirrhosis hepatis in advanced Wilson’s disease. (**A**): Severe fibrosis is seen on elastography (**B**): Advanced cirrhosis with ascites seen on T2-weighted MRI scan.

The patients’ demographic data and results of the laboratory tests were collected from electronic medical records. We also calculated the fibrosis score with serological markers, online calculator, using the APRI, FIB-4, MELD-Na, Child–Pugh, NAFLD fibrosis-, BARD-score and, in some rare cases, MRI and/or liver biopsy were also performed (Figure 2).

### 2.4. Statistical Analysis

We report the continuous variables as mean ± standard deviation (SD) and categorical variables as number (n) and percentage (%). The continuous variables were compared between patient groups using a *t*-test. We used Fisher’s exact test to compare the distributions of categorical variables between patient groups and calculated odds ratios (OR) along with 95% confidence intervals (CI). Pearson’s correlation coefficient (r) was calculated to describe colinearity between continuous variables. Multivariable and univariable linear regression models were constructed to evaluate the associations between UEFF, the dependent variable, and clinical or laboratory indices as predictor variables. For regression models we report the coefficient of determination (R2) and the *p*-value.

The *p* < 0.05 was used as the threshold of statistical significance. The statistical analysis was performed in R version 4.3.3. (www.r-project.org, accessed on 29 February 2024).

## 3. Results

Altogether, 53 patients (18 females and 35 males) with WD were examined by SWE. Ultrasound-based parameters, TAI, and TSI for quantification of liver steatosis and SWE-based liver stiffness (LS) measurement were assessed. Regarding sex differences, female patients had significantly lower body weight and height (*p* < 0.001 and *p* = 0.036, respectively), while no significant difference was observed in BMI (*p* = 0.87) or ultrasound-based parameters (TAI, TSI, and SWE-based liver stiffness). Furthermore, the high standard deviations observed in liver function tests (e.g., ALAT, GGT) across both sexes reflect the substantial clinical heterogeneity of our cohort. This variability is consistent with the full clinical spectrum of Wilson’s disease, ranging from asymptomatic or neuropsychiatric phenotypes to advanced hepatic involvement.

Summary of the patient’s demographic, laboratory, and imaging data is shown in Table 1. The patients are typically from a relatively younger population, with a BMI ± SD of 23.71 ± 4.15 kg/m^2^. Among them, only two patients have type 2 diabetes mellitus, 13 of 53 patients (24.5%) are overweight (BMI: 25–29.9 kg/m^2^), and 6 of 53 patients (11.3%) are obese (BMI ≥ 30 kg/m^2^).

Liver fat content was measured by quantitative ultrasound (QUS) method in patients with Wilson’s disease—both with and without impaired glucose tolerance, type 2 diabetes mellitus, or hyperlipidemia—across lean, overweight, and obese individuals. Among our patients with Wilson disease, 37 (69.8%) had no steatosis (S0), 14 (26.4%) mild (S1), and 2 (3.8%) intermediate (S2) stage, and none of them had severe (S3) steatosis based on UEFF calculation.

We also used the Hepatic Steatosis Index for MAFLD parallelly to the US measurements, but we have not found significant correlations between this score and our ultrasound measurements.

**Figure 3:**
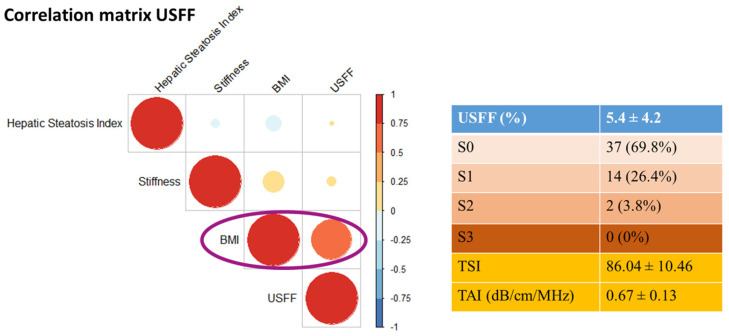
Correlation matrix of liver steatosis measures in patients with Wilson’s disease. The body mass index (BMI) demonstrated a strong positive correlation with ultrasound-estimated fat fraction (UEFF); however, it did not correlate with liver stiffness or the clinical indices used to assess liver steatosis. The different colored dots represent the magnitude and direction of the correlations between the variables. The color bar on the right side of the plot represents the Pearson correlation coefficient.

**Figure 4:**
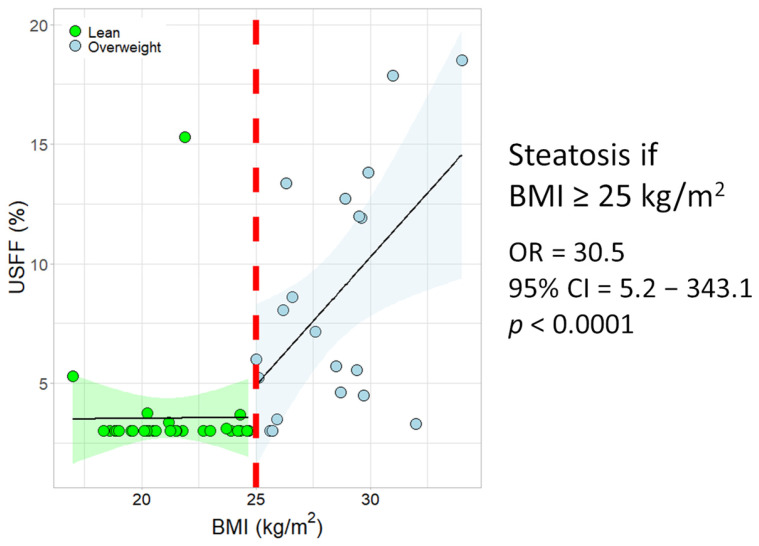
Scatter plot of body mass index (BMI) and ultrasound-estimated fat fraction UEFF) in Wilson’s disease patients. The regression line (solid line) did not indicate an association between BMI and UEFF in lean patients (green shaded confidence interval). Meanwhile, above a threshold of 25 kg/m^2^ (red line) in overweight individuals, the regression line (blue shaded confidence interval) displayed a positive and significant association between BMI and UEFF. Only one patient in the lean group had liver steatosis; all other cases of steatosis belonged to the overweight group (OR = 30.5, 95% confidence intervals = 5.2–343.1, *p* < 0.0001).

Meanwhile, we found a significant overall correlation between BMI and UEFF (r = 0.626, *p* < 0.0001) (Figure 3). The BMI also showed a significant negative correlation with serum ceruloplasmin (r = −0.486, *p* < 0.0004) and copper (r = −0.41, *p* < 0.004) levels. Among patients with a BMI < 25 kg/m^2^, only one had liver steatosis (OR = 30.5, 95% CI = 5.2–343.1, *p* < 0.0001). When the patients were divided into lean (BMI < 25 kg/m^2^) and overweight (BMI ≥ 25 kg/m^2^) groups, and regression analysis was performed to identify risk factors of liver steatosis, we found a significant association between BMI and UEFF R2 = 0.288, *p*< 0.015) in the overweight, not in the lean group (R2 = 0.0065, *p* = 0.966) (Figure 4).

WD patients with normal BMI had no significant steatosis according to the UEFF measurement, while patients with more than 25 kg/m^2^ had elevated fat fraction during the US measurement, with positive correlation.

Based on LS values measured with 2D-SW, 41 (77%) patients did not have significant liver fibrosis (F0 or F1), 5 (9%) patients had moderate (F2) and 4 (8%) advanced (F3) fibrosis, while 3 (6%) patients had cirrhosis after applying the cut-off values reported by Paternostro et al. [24].

**Figure 5:**
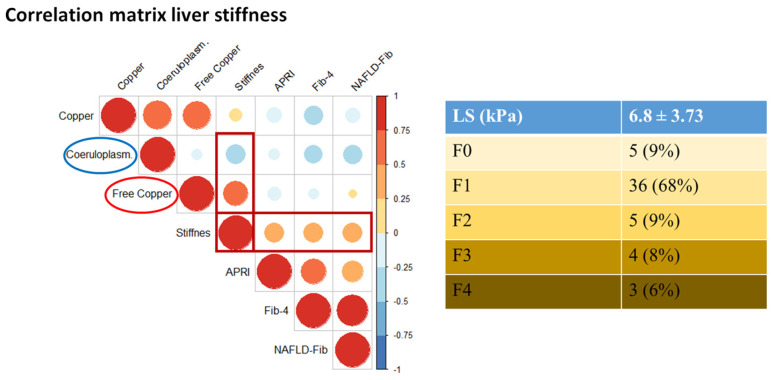
Correlation matrix of metrics of liver fibrosis in Wilson’s disease patients. The liver stiffness (LS) was measured using 2D shear wave elastography (2D-SWE), and it showed a positive correlation with calculated free copper and a nearly significant negative correlation with ceruloplasmin levels. The clinical indices of liver fibrosis (APRI, Fib-4, and NAFLD fibrosis index) showed a moderate correlation with LS. The different colored dots represent the magnitude and direction of the correlations between the variables. The color bar at the right side of the plot represents the Pearson correlation coefficient.

The LS was in moderate correlation with FIB-4 (r = 0.306, *p* < 0.03), NAFLD fibrosis index (r = 0.336, *p* < 0.02), and APRI (r = 0.31, *p* = 0.0857). However, we found a significant positive correlation between LS and calculated free copper levels (r = 0.52, *p* < 0.0022), which was observed in conjunction with a nearly significant negative correlation (r = −0.32, *p* < 0.0762) between ceruloplasmin and LS (Figure 5).

Several WD patients under our care have already had multiple elastography measurements. Summary of the patient’s demographic, treatment and imaging data is shown in Table 2. On appropriate treatment, their liver state did not generally worsen with age, according to the fibrosis score.

**Figure 6:**
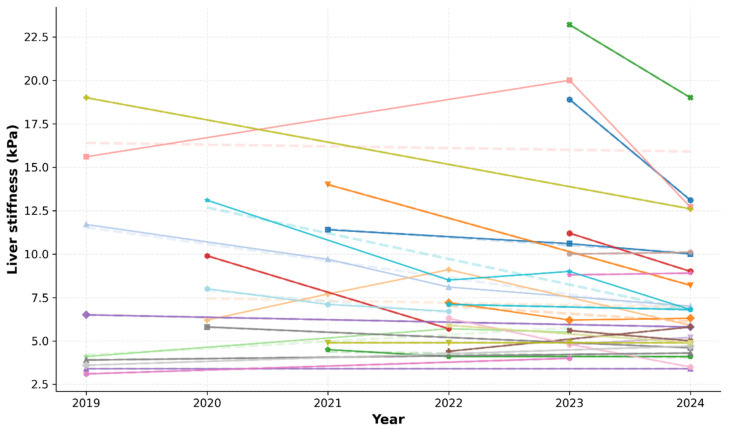
Liver stiffness values between 2019 and 2024 from retrospective data. Longitudinal assessment of liver stiffness (LS) values. Individual scatter plot representing all recorded LS measurements for each patient. Individualized trend lines illustrate the progression or stabilization of LS parameters over a five-year follow-up period for each participant.

During the years (2019–2024, median time: 32 months, range: 8–78 months), the liver stiffness parameters were typically constant or improving with appropriate treatment of D-penicillamine, trientine, or zinc acetate (*p* = 0.468, one-sided unpaired *t*-test). In most patients, the fibrosis stage did not change substantially; six patients showed improvement and moved to a lower fibrosis stage, while two patients progressed to a more advanced stage (Figure 6).

Nevertheless, it should be noted that comparisons between measurements obtained using different elastography methods are not entirely accurate.

Among the WD patients, 36 have been treated with D-penicillamin, 12 with zinc acetate, and 5 with trientine dihydro- or tetrahydrochloride.

Based on the clinical parameters (symptoms, laboratory data—serum bilirubin, transaminase enzyme levels, synthetic capacity parameters and urinary excretion), calculated fibrosis markers, and measured elastography values, the treatment was appropriate, and their liver elasticity, according to the measured stiffness values, was unchanged in most patients or showed improvement in some patients (illustrated by the trend lines).

In case of progression, other reasons must also be taken into account (e.g., reduced glucose tolerance, metabolic steatotic disorder, associated other liver disease, loss of compliance).

Based on our calculations, the values indicating measured steatosis showed significant association with the BMI above a threshold of 25 kg/m^2^ and fibrosis were mostly correlated with liver enzymes and triglyceride values.

In two cases, progression of liver disease was observed with elevating liver parameters and in one case with decompensation signs and symptoms. One of them had a successful orthotopic liver transplantation.

## 4. Discussion

This is the first study to evaluate the status of liver disease by assessments of liver fibrosis and steatosis in a significant proportion of Wilson’s disease patients in Hungary using non-invasive methods.

In advanced liver cirrhosis, the clinical signs are clear, but in the compensated stage, diagnosing cirrhosis is often more challenging. The assessment of liver fibrosis in chronic liver diseases has a significant prognostic value. Liver biopsy is the gold standard for the diagnosis of liver cirrhosis and fibrosis. However, biopsy is an invasive procedure that could have various complications, such as bleeding [20].

In cases of fulminant hepatitis, the confluent necrosis observed in WD does not have distinguishing features from other causes of massive hepatic necrosis. The steatosis without any other histological alteration could be the first manifestation of WD, making it difficult to make a correct diagnosis.

Increasingly accurate ultrasound-based elastography measurements provide important support in non-invasive state assessment during the follow-up of WD patients, but concerning the diverse, colorful histological manifestations and variable extent of the individual histological abnormalities, it is understandable that determining the exact elastography cut-off values is challenging.

Non-invasive diagnostic methods provide significant assistance in all of these areas. Liver biopsy is rarely performed.

Various serum calculators and non-invasive ultrasound- or MR-based measurements assist us in determining the stages of liver fibrosis and steatosis. Nehring P et al. published a descriptive review of available elastography techniques and the results of the most recent studies of elastography of the liver in patients with Wilson’s disease [27].

According to the etiology of liver disease, liver stiffness values vary for the same stage of fibrosis. Higher values can be seen in autoimmune hepatitis, compared to Wilson’s disease and HCV groups (16.15 ± 7.23 kPa, 8.30 ± 0.84 kPa, and 7.43 ± 1.73 kPa, respectively, according to the data of 90 children with biopsy-confirmed chronic liver diseases) [28,29].

In chronic liver diseases, liver stiffness is increased, primarily because of liver fibrosis, but other factors, such as intrahepatic deposits, may also be involved. In chronic liver diseases, liver stiffness is increased, primarily because of liver fibrosis, but other factors, such as intrahepatic deposits, may also be involved. Our results demonstrated a significant positive correlation between LS and calculated free copper levels (r = 0.52, *p* < 0.0022), while previous studies in pediatric populations [30,31] found that LS decreased parallelly with increased urinary copper excretion during initial treatment. Our results in a largely treated adult cohort suggest that residual free copper continues to impact tissue stiffness.

In a larger study, based on 188 WD patients’ data, Paternostro et al. conclude that transient elastography and non-invasive fibrosis scores are useful to identify cirrhosis in patients with WD with a cut-off value of 9.9 kPa for liver cirrhosis, which is lower than cut-offs reported in other etiologies [31,32]. We used this cut-off value as well to define advanced fibrosis. Seven of our patients were categorized in this more severe stage.

Several data sets are available regarding the cut-offs for cirrhosis in chronic hepatitis C [31,33,34]; values in the literature range between 10 and 17.6 kPa. However, the most recent guidelines prefer rule-out and rule-in ranges, usually in combination with other non-invasive markers.

Regarding non-invasive laboratory-based scores, APRI and FIB-4 were both useful to rule out advanced liver disease rather than diagnosing cirrhosis. They could, therefore, in addition to liver stiffness measurement, help identify/rule out cirrhotic WD patients.

Among the mathematical calculation models, APRI and FIB-4 scores were found useful for patients with WD, with a proposed cut-off value of <1.5 for APRI and <3.25 for FIB-4, with a specificity of 93% and 95%, respectively, to exclude liver cirrhosis [24,31]. We have found weak, but significant correlation, between these scores and elastography measurements.

In Wilson disease excess free copper promotes oxidative stress, with activation of Fenton reaction, resulting in various changes in tissue with mitochondrial injury, cell damage, hepatocellular swelling, lobular hepatitis, and hepatocyte necrosis. We observed a nearly significant negative correlation between ceruloplasmin and LS (r = −0.32). LS elevations in WD may reflect active copper-induced inflammatory activity rather than structural fibrosis alone. Therefore, improvement of elastographic data could also be explained by either fibrosis regression or decreased inflammation. This distinguishes WD from other etiologies where LS more strictly refers to collagen deposition.

Regrettably, we cannot definitively address this issue due to lack of histological confirmation of hepatological involvement, as liver biopsy is no longer strictly necessary for the diagnosis of WD.

In advanced WD with cirrhosis, hypoalbuminaemia and low ceruloplasmin levels can alter the distribution of copper fractions. Specifically, reduced protein binding may lead to an apparent increase in calculated free copper, creating a potential analytical overestimation unrelated to true copper toxicity. This also complicates the interpretation of LS–copper correlations in decompensated disease. To obtain a more accurate clinical picture in the advanced stage we accounted for these parameters in our calculation.

While characteristic ultrasound features of the liver parenchyma are instrumental in diagnosis of WD, the degree of hepatological involvement often shows a poor correlation with actual histopathological damage. Lie et al. categorized liver echo patterns of WD according to the B-ultrasound and correlated the degree of hepatic injury with different liver echo patterns based on transient elastography (TE) and Sound Touch Viscoelastography (STVi). Their findings suggest that combining these methods allows for a more reliable grading of hepatic injury in WD patients, effectively bridging the gap between imaging patterns and functional liver damage [35].

Several studies tried to find a correlation between elastography measurement and liver fibrosis stage. Sini et al. have found increased values proportionally with progression and histological fibrosis score, with a proposed cut-off value of 6.6 kPa for significant fibrosis and 8.4 for advanced fibrosis [36]. Others have found no clear correlation between SWE measurement and METAVIR score. The lack of correlation in WD might be the consequence of the varying liver involvement patterns, micro- and macrovesicular steatosis, glycogenation of nuclei and mitochondrial changes, deposition of copper salts, portal and periportal inflammation [37].

Among our WD patients, most of them were treated for several years (51/53) and only two of them were recently diagnosed. Therefore, we could not see such a marked decrease in liver stiffness values during that time period, than in the case of recently diagnosed WD patients. Long-term follow-up would be meaningful.

In a Turkish study, treated pediatric patients with WD were evaluated for parenchymal changes in both the liver and pancreas related to copper accumulation, using ultrasound-based assessment [38]. The authors found that transient elastography provided valuable information on early alterations in tissue stiffness, thereby enabling objective monitoring of WD patients who require lifelong follow-up. Based on these findings, we advocate for the integration of elastographic assessments into the routine clinical follow-up of WD patients.

Diverse data could be found regarding the use of fibrosis markers in WD patients [31,39,40]. Limitation factors can be the relatively low frequency of this genetic disorder, with limited data and a mostly retrospective study nature.

For the assessment of steatosis of the liver, the attenuated coefficient (ATT) has been recently used. J Wang et al. has found that ATT could be a valuable tool as a non-invasive index for evaluation of liver steatosis in children and adolescents with WD, with a good clinical applicative value [37], with a better diagnostic performance compared to APRI, FIB-4 and SWM in the assessment of early liver involvement in their WD population and its diagnostic efficacy was improved when combined with other indices.

During the conventional two-dimensional B-mode abdominal US examination visualizes the whole liver, shear-wave elastography can be performed under visual inspection, avoiding blood vessels and other structures. Another advantage of TAI and TSI is that these are real-time measurement methods.

Previously in MASH/MASLD, we confirmed that TAI and TSI could be used successfully to diagnose and estimate the severity of hepatic steatosis in routine clinical practice with an excellent reproducibility for TAI (ICC = 0.95) and moderate reproducibility for TSI (ICC = 0.73) during interobserver analysis [25].

We used the same methods for the evaluation of steatosis in WD patients. A central finding of our results was the differential behavior of hepatic steatosis based on UEFF measurements by metabolic status. While steatosis can be an early manifestation of WD regardless of weight, our data revealed that significant steatosis was almost exclusively present in patients with a BMI over 25 kg/m^2^. It seems obvious that copper deposition also plays a role in steatotic transformation of the liver. Among patients with a normal BMI, only one individual had steatosis, resulting in a striking Odds Ratio of 30.5.

This means that hepatic steatosis is increasingly driven by metabolic factors rather than copper-induced lipid accumulation alone. The significant correlation between BMI and UEFF (r = 0.626, *p* < 0.0001) in the overweight group, but the complete absence of this correlation in the lean group (R^2^ = −0.034), indicates that weight management is a critical, independent component of liver health in WD patients.

Our cohort with a median follow-up of 32 months, demonstrated notable LS stability. We observed that under appropriate treatment LS parameters remained constant or improved in the majority of patients. This confirms the value of SWE as a follow-up method. In case of elevation in LS in a previously stable patient should trigger an investigation into treatment compliance or the development of metabolic comorbidities.

A limitation of our study is the relatively small sample size, the short-term follow-up period, and the fact that it was a single-center retrospective case–control study. Furthermore, histological correlation—the diagnostic gold standard—was available in only a limited number of cases to confirm the reliability of our non-invasive assessment methods. In absence of systematic histological confirmation, the current findings should be interpreted as preliminary observations. Nevertheless, the observed correlations provide a basis for further prospective studies involving larger cohorts where non-invasive markers can be more rigorously validated against clinical outcome.

The data from these few patients do not permit more definitive conclusions to be made.

In the future, we plan to follow up our patients long-term by non-invasive radiological and serological assessment in everyday practice and patient care.

## 5. Conclusions

Using non-invasive radiological measurements, we are able to obtain an increasingly accurate picture of the current state of the liver during follow-up, assessing the progression of the underlying disease. By comparing this data, we gain insights into how frequently we should schedule follow-up appointments for the patient and when we should prepare for liver transplantation. Evaluating all information together with free copper level determinations and urinary copper excretion tests provides a more precise understanding of the disease, and we can assess the patients’ current condition.

Overall, our research highlights the importance of regular and appropriate monitoring of Wilson’s disease. In addition to clinical and laboratory examinations, serological and ultrasound methods may play an increasingly important role, supplemented by histological assessment if it is necessary.

## Figures and Tables

**Table 1:** Demographic and clinical characteristics of the study cohort.

		Female (*n* = 18)	Male (*n* = 35)
Mean age (yrs)	40.5 ± 13.1	43.2 ± 15.2	39.1 ± 12.0
Weight (kg)	75.3 ± 16.0	65.0 ± 11.0	80.4 ± 15.7
Height (cm)	173.9 ± 25.3	166.2 ± 6.3	177.7 ± 30.1
BMI (kg/m^2^)	23.7 ± 4.2	23.6 ± 4.5	23.8 ± 4.0
LDL cholesterol (mmol/L)	3.0 ± 0.8	3.3 ± 0.8	2.9 ± 0.8
Triglyceride (mmol/L)	1.5 ± 1.3	1.5 ± 1.1	1.6 ± 1.4
Bilirubin (µmol/L)	14.9 ± 12.1	12.1 ± 5.2	16.3 ± 14.3
ASAT (U/L)	29.8 ± 17.2	28.7 ± 16.3	30.4 ± 17.8
ALAT (U/L)	46.9 ± 62.1	44.7 ± 64.9	48.0 ± 61.6
GGT (U/L)	48.2 ± 51.6	57.6 ± 76.1	43.3 ± 33.3
ALP (U/L)	86.7 ± 26.8	82.7 ± 30.4	88.7 ± 25.0
Serum copper (µmol/L)	6.2 ± 4.8	8.50 ± 5.7	5.14 ± 4.3
Serum ceruloplasmin (g/L)	0.14 ± 0.1	0.18 ± 0.1	0.13 ± 0.1
INR	1.17 ± 0.1	1.18 ± 0.1	1.16 ± 0.1
Platelet (G/L)	239.2 ± 72.6	253.9 ± 65.4	231.6 ± 75.8
Obesity (*n*)	6	2	4
Overweight (*n*)	13	5	8
Type 2 diabetes mellitus (*n*)	2	2	0
APRI	0.29 ± 0.16	0.31 ± 0.15	0.27 ± 0.17
FIB-4	0.84 ± 0.62	0.85 ± 0.59	0.92 ± 0.65
MAFLD	−3.13 ± 1.49	−2.94 ± 1.50	−3.25 ± 0.90
BARD	1.87 ± 1.55	2 ± 0.89	1.74 ± 0.89
**US parameters**			
UEFF (%)	5.4 ± 4.2	3.9 ± 1.6	6.1 ± 4.9
TAI med ± SD	0.66 ± 0.12	0.65 ± 0.12	0.68 ± 0.13
TSI med ± SD	85.7 ± 10.3	83.4 ± 11.2	87.1 ± 9.7
LS (kPa)	6.8 ± 3.7	7.0 ± 3.7	6.5 ± 3.4

**Table 2:** Demographic and clinical characteristics of the follow-up cohort.

	Mean ± SD
Mean age (yrs)	40.5 ± 13.9
Weight (kg)	74.9 ± 15.6
Height (cm)	176.7 ± 11.1
BMI (kg/m^2^)	23.8 ± 4.2
Treatment duration (years)	18.3 ± 10.3
DPA therapy (*n*)	43 pts
Trientine therapy (*n*)	7 pts
Zinc acetate therapy (*n*)	8 pts
Follow-up period (months)	36.6 ± 22.5
Liver stiffness at baseline (kPa)	8.1 ± 8.23
Liver stiffness at follow-up (kPa)	4.92 ± 6.86

## Data Availability

The research data are available upon request.
